# Utility of SPECT/CT in Sentinel Lymph Node Detection in a Case of Vulvar Carcinoma

**DOI:** 10.4274/Mirt.161

**Published:** 2013-12-10

**Authors:** Chidambaram Natrajan Balasubramanian Harisankar, Bhagwant Rai Mittal, Anish Bhattacharya, Lakhbir Kaur Dhaliwal

**Affiliations:** 1 Postgraduate Institute of Medical Education and Research, Nuclear Medicine and PET, Chandigarh, India; 2 Postgraduate Institute of Medical Education and Research, Obstetrics and Gynaecology, Chandigarh, India

**Keywords:** Sentinel lymph node biopsy, Single –Photon Emission Computerized Tomography, Computeized Tomography, X ray, lymphoscintigraphy, Vulvar cancer

## Abstract

Minimally invasive sentinel node biopsy is associated with significantly less morbidity and has been evaluated in several studies in patients with gynecologic malignancies. Accurate identification of the sentinel lymph nodes is possible in most of the patients. Hybrid SPECT/CT is a newer modality which has been shown to improve the localization of the suspicious lesions and also provide anatomical information of the involved lymph nodes. We report the utility of hybrid SPECT/CT in localization of sentinel lymph node in a case of vulvar cancer and its impact on patient management.

**Conflict of interest:**None declared.

## INTRODUCTION

The use of lymphatic mapping and sentinel node detection for cutaneous melanoma and breast cancer has been extensively investigated, and now minimally invasive sentinel node biopsy, which is associated with significantly less morbidity, has successfully replaced complete nodal dissection for nodal staging. This minimally invasive approach has been evaluated in several studies in patients with gynecologic malignancies, which have shown it to be capable of accurately identifying sentinel nodes. SPECT/CT provides both anatomical and functional information about the sentinel lymph node. We report the utility of hybrid SPECT/CT in localization of sentinel lymph node in a case of vulvar cancer.

## CASE REPORT

**Figure 1:**
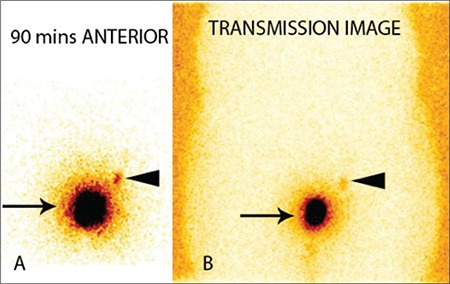
Static planar images acquired in anterior view without (A) andwith transmission source (B). Intense tracer activity at the site of injection(arrows). Faint tracer activity in the left inguinal region (arrow heads) isnoted at 90 minutes

**Figure 2:**
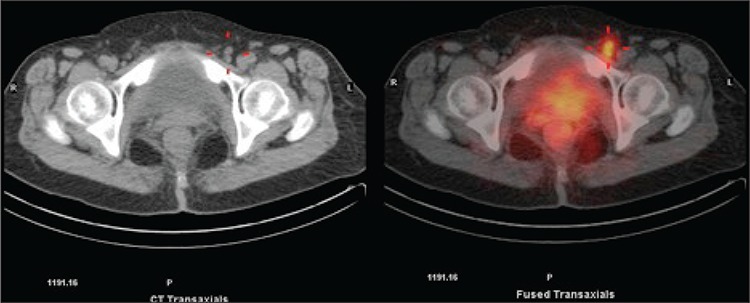
Hybrid SPECT/CT images of the pelvis showing focal tracer uptake in small left inguinal lymph node (Triangulation). Also noted is intense traceractivity in the vulva (site of injection)

A 40 year old female presented with bleeding vulvar ulcer on the right side (T1 stage tumor). A biopsy from the edge of the ulcer revealed squamous cell carcinoma. The patient was planned for definitive surgery. A sentinel lymph node dissection was planned. Methylene blue dye was not used. Technetium-99m labeled unfiltered sulfur colloid was used and injection performed on the day of the surgery. Radiotracer in a dose of 100 microcurie each was injected at four sites around the tumor. Multiple static images were acquired till the visualisation of the sentinel lymph node. A hybrid SPECT/CT was performed immediately after visualisation of the sentinel lymph node. A focus of tracer uptake was noted in the planar image in the left inguinal region at 90 min after the injection. SPECT/CT localized the tracer uptake in a 1.2 cm sized left inguinal lymph node (Figure 1 and 2). The exact anatomical location of the lymph node and its relation with the adjacent structures were also defined by SPECT/CT. The left inguinal lymph node was successfully localized with an intra-operative probe and was subjected to histopathological examination. No metastases were noted in the lymph node on pathological examination. An unnecessary lymphadenectomy was avoided and the patient is on follow up without evidence of disease progression.

## DISCUSSION

Finding of positive sentinel nodes at surgery significantly influences staging and subsequent clinical management. In particular, a sentinel lymph node free from tumor metastasis would exclude tumor spread to the at-risk regional lymphatic basin. Although it is possible that a negative sentinel lymph node corresponds to metastatic involvement of a second-tier lymph node, this occurrence is very rare, especially when the primary tumor is in an early stage of growth. The sentinel node concept remains thus valid in most patients.

Studies have shown the efficacy of sentinel node mapping in cutaneous melanoma (1,2). Sentinel node mapping has been shown to be effective in gynecologic malignancies (3,4) including vulvar cancer (5,6). The accurate localization of sentinel nodes for tissue sampling has become increasingly important for clinical management. Preoperative SPECT/CT lymphoscintigraphy is ideal in this regard because it can map unpredicted lymphatic drainage pathways within the complex pelvic anatomy (7,8). SPECT/CT is able to achieve the precise anatomic localization of sentinel nodes on cross-sectional imaging, especially in relation to known anatomic structures such as the femoral or saphenous vein, information that is critical in planning the surgical approach. SPECT/CT also increases the sensitivity of sentinel node detection (9). A node close to the injection site can also be masked as the result of strong activity from the injection site (ie, the “shine through” effect). SPECT/CT avoids this problem and improves the detectability of the lesion (10). SPECT/CT has the potential to revive the usage of sentinel lymph node dissection.
